# US Farm Animal Welfare: An Economic Perspective

**DOI:** 10.3390/ani9060367

**Published:** 2019-06-18

**Authors:** Glynn T. Tonsor, Christopher A. Wolf

**Affiliations:** 1 Department of Agricultural Economics, Kansas State University, Manhattan, KS 66506, USA; 2 Department of Agricultural, Food and Resource Economics, Michigan State University, East Lansing, MI 48824, USA; wolfch@msu.edu

**Keywords:** consumer demand, economics, farm animal welfare, producer perspective

## Abstract

**Simple Summary:**

Farm animal welfare is one of the more controversial and complicated topics facing modern agriculture. To proceed forward, a sound understanding of multiple economic aspects must be appreciated. This article provides a short summary of these economic perspectives with a focus on the US situation.

**Abstract:**

The topic of farm animal welfare (FAW) is both complex and controversial, and inherently involves expertise and views from multiple disciplines. This article provides a summary of economic perspectives on FAW issues in the United States. Practices related to FAW can occur through legal, market or voluntary programs. FAW is not a primary driver of US food demand but negative press has industry-wide effects. Aligning FAW supply and demand can be facilitated through labeling, education, and voluntary programs, but all have pros and cons.

## 1. Introduction

US livestock, dairy and poultry producers have faced increasing scrutiny of production practices perceived to be related to farm animal welfare (FAW) in recent years [1,2,3,4]. FAW is no longer a fringe issue—but it is also not the primary driver of US food demand. Consumers expect a safe, reliable, and affordable food supply, which US farmers have and will continue to provide. The general US population has little connection to modern commercial agriculture and, therefore, essentially no context to accurately understand production practices. Surveys consistently reveal a high level of trust and respect for farmers and maintaining that trust is of utmost importance to ensure market access and avoid unnecessary regulation. 

Livestock producers are the primary caretakers of farm animals and their decisions regarding housing systems, feed type, and health management directly impact FAW [5]. In economic terms, livestock producers are the suppliers of farm animals, providing meat, milk, egg, and other products demanded by consumers [6]. Production economics can help us understand the producer’s situation, reflecting the complex set of economic incentives and constraints they face. Moreover, recognizing behavioral aspects of how livestock producers view FAW and what attitudes they hold regarding FAW further this understanding [7].

The primary motivation for producers to recognize and respond to public and consumer concerns, perceptions and demands for FAW-related production practice changes is maintaining the social license to produce. Social license is a concept that relates to the public trust in the production system. Without social license, regulation of some sort would follow. In general, producers wish to avoid regulation as it tends to increase costs—particularly in heterogeneous industries when a rigid standard is applied.

Given a great deal of research on consumer perceptions and demand for FAW has been carried out, there are many general conclusions and lessons that can be drawn with implications for farm practices [8]. This article provides a summary of economic perspectives on FAW issues in the United States. Understanding the economic situation facing livestock producers and reflecting on the myriad of formal and informal signals they receive regarding production practices can elevate the overall understanding regarding current and future FAW [6]. This article is structured as a series of observations supported by the economics literature to spur more efficient decision-making and subsequently improve FAW management and policy outcomes. 

## 2. Methods of FAW Practice Change

Over time, FAW-related production practices adjust as livestock producers respond to an array of signals which can broadly be viewed as two primary methods of change: Market and legal [9]. In the market method, FAW adjustments occur from livestock, dairy or poultry producers seeking to fulfill a perceived market opportunity. Market-driven changes occur as retailers, such as grocers and restaurant chains, insist on practices that they can market at the farm level. In some cases, the retailers are responding to pressure from consumer groups, but often they are seeking to increase corporate image. One example is voluntary enrollment in the 5-step Global Animal Partnership program, widely associated with Whole Foods Market (a US-based grocer primarily selling natural and organic products) [10]. A second example is enrollment in a United States Department of Agriculture (USDA) Process Verified Program [11]. Other examples include partial shifts toward swine production without use of gestation stalls and egg production without use of laying hen cages. In each case, voluntary adjustment by producers reflects a segment of livestock being produced in a different way, corresponding with private efforts to align the supply of livestock products carrying a FAW claim with higher livestock prices offered by upstream livestock buyers. 

Perhaps the most contentious method of farm animal welfare-related practice change is legal. Changes in legislation or ballot initiatives result in legally binding consequences for how animals are reared in a specific geographic region [12]. A high-profile ballot example is the 2008 passage of Proposition 2 in California [13], while the US 28-h transportation rule [14] is an example of binding legislation. A common concern with the legal avenue is that “unfunded mandates” follow—producers are required to adjust practices without corresponding monetary benefits that help offset associated adjustment costs [15]. This “vote-buy” gap is core to contention of the legal avenue [16]. A main benefit of the legal avenue is establishment of clear minimum standards impacting all covered producers. Ballot initiatives can be attractive to activist groups. These initiatives are often written generically about farm animal space and natural behavior allowances. Ballot initiatives do not specify detailed system practices and the method to which they are implemented can be very important when determining actual farm effects. This lack of detail is also a source of great uncertainty for farmers who must make investment decisions. In some cases, legislation is used to avoid a ballot initiative and may be a useful alternative if the animal industries can be involved in the process. 

Either the market or legal method can be the impetus for voluntary programs adopted by producers. These programs might realize premiums for farmers—or at least avoid market discounts. It is important to note that these programs are often motivated by the desire to avoid legal mandates or maintain market access. The primary advantage of a voluntary program can be input from farmers, industry and consumers to arrive at a set of standards that allow for a flexible farm response. As there is a great deal of variation in farm size, methods and practices, there are a number of ways for farmers to achieve FAW standards, which voluntary programs can enable.

## 3. A Minority of Residents Will Truly Pay More for FAW Change

In an examination of 11 different food values, Lister et al. (2018) found the importance of FAW was very low for US consumers [17]. Specifically, shares of importance on four different products were consistently 4–5%: Ground beef (5.2%), beef steak (4.6%), chicken breast (4.1%), and milk products (4.8%). Conversely, the relative importance of price was consistently around 20%, conveying that, on average, price is four-times as important to US consumers. 

Consistently, the vast majority of consumers have rated price as the most important factor. Consumers that profess to higher willingness-to-pay (WTP) for animal welfare-related products tend to be older, female, and have higher incomes. However, all hypothetical WTP estimates should be interpreted in the context of hypothetical bias. That is, talk is cheap and saying you would pay more without the requirement to follow through is costless to the research subject. Further, when respondents believe that a specific answer reflects favorably upon them, they may choose the most flattering rather than most correct answer. This is referred to as social desirability bias and likely increases the stated desire for FAW change [18].

When making a purchase of almost any product, most consumers primarily focus on price. The reality is that budget constraints make concern over production practices a luxury for many consumers. A relatively small percentage of the US public cares a great deal about animal welfare to the extent it alters their actual food purchases. However, small, passionate groups drive discussions and often practice changes in many areas. Consumers that are most concerned with animal welfare when making food purchases tend to be older, wealthier, and female [15,19]. This is not to say that others do not consider animal welfare aspects, but they tend to be of secondary importance to most consumers.

## 4. FAW Is Confounded with Other Food Topics

With respect to FAW, public perceptions vary depending on species and the way in which questions are asked. For example, framing a practice in positive light will result in different responses than when it is framed in a negative light. However, some general patterns hold. First, food safety is an issue that overwhelms all others when it is present or perceived. Consumers have no interest in food products if safety is in question. This makes claims or labels that imply food safety issues particularly damaging. US agriculture has a stellar record of supplying safe food and maintaining this trust is paramount. Food safety is often conflated with technology, farm size, and environmental issues. Tonsor, Olynk, and Wolf (2009) found farm size attributes to be a substitute—from the perspective of US pork consumers—for FAW-oriented production practices [20].

## 5. FAW Negative Press Matters

When undercover videos or other evidence of animal welfare issues on commercial farms surface, they are widely viewed and affect public perceptions of the entire industry. Tonsor and Olynk (2011) found media attention to animal welfare had a small, but statistically significant impact on US pork and poultry demand [21]. The main economic impact was consumer expenditure reallocated to non-meat products, rather than spillover to competing meats. Contesting ballot initiatives has also been shown to have negative market repercussions, as it increases public attention on perceived farm animal welfare issues [19,22]. More recently, disputes over “ag gag” laws—that seek to limit the ability of opponents to take undercover videos and pictures—have led some to infer that animal agriculture has something to hide. Because negative media affects the entire industry, there is reason for programs that maintain social trust.

## 6. Why Don’t Producers Maximize FAW?

Ultimately, a basic question arises in FAW discussions—why livestock producers simply do not “do more”. A situational summary provided by Lusk and Norwood is useful in considering this question [6]. At the extreme, some ask why producers do not make a focused effort to maximize FAW. While improving FAW is a worthwhile goal, it is critical to appreciate that livestock producers are no different than other individuals—they face trade-offs. Any investment or adjustment in effort designed to improve FAW comes at the expense of an alternative investment or effort that is forgone [23]. Stated differently, difficult decisions are regularly made in rearing livestock to meet multiple goals, including, but certainly not limited to, FAW, safety of animal agriculture products, environmental impact, livestock disease resistance, and firm profitability. The key point is that while improvement in FAW is certainly worth discussing and a noble goal, there is no free lunch and tough decisions always must be made.

## 7. Ways Forward for US Animal Agriculture

So, what are some solutions for the farm animal industry to maintain social trust with respect to perceived animal welfare issues? Assuming that farmers would prefer to avoid formal regulation, options include labeling, education, and voluntary certification programs. Process labels describe how an animal was raised, crops were grown, or ingredients were transformed. Often these labels focus on what was not done or used, as is the case for “hormone-free” and “GMO-free” labels. Advantages of process labels include that they provide information, may enhance trust, and can assist in segmenting the market [24]. Disadvantages include potential information overload (when additional information can be distracting and complicate decisions), confusion, and elevated food safety and risk perceptions [25]. Labels communicating a production technology often induce a negative reaction, resulting in decreased demand for safe products. For example, “contains” has a negative connotation while “free of” a positive connotation. There are also labeling costs to consider, which results in less support for labeling [26].

Information changes consumer preferences. This is particularly true when evaluating food attribute trade-offs [3]. Educational programs are often put forward as a solution because few people have context for production agriculture (~2% of US citizens are farmers). There is some evidence that educational programming, such as Breakfast on the Farm, works to enhance public perception and trust. However, people must want to be educated. There is also some evidence that educational programs can work against intended goals with lack of context and understanding of production agriculture [27]. Educational programs can highlight that many valued practices are already supplied by current commercial agriculture [28]. Another aspect of education to consider is that consumer FAW concerns are about the production process, rather than the product itself. That is, the concern is with the animal rather than the meat, milk, or eggs. Lack of recognition about this often leads to producers and consumers talking past each other in an unproductive manner. Education should not be conflated with activism or it runs the risk of inducing a negative reaction.

Finally, voluntary programs can assist in maintaining public trust. Program effectiveness depends largely on certification. Research has shown that certification or verification is fundamental to public acceptance of animal welfare programs [18]. While people often profess to lack trust in the government, research reveals that consumers often do not trust industries to police themselves and prefer USDA certification [29]. Consumers also have more faith in programs where violations result in real consequences.

## 8. Conclusions

Viable food animal agriculture requires public trust. Social concerns related to production agriculture are unlikely to diminish in future years. Awareness and incorporation of these concerns in production practices and marketing will help ensure social trust, limit restrictive regulation, and ultimately improve the well-being of animals and people alike. The current literature suggests that FAW, while not the key driver of demand, has important market effects. The US livestock, dairy and poultry industries should continue to move to drive the conversation and be involved in any policy discussions related to potential regulation. Education, labeling, and voluntary programs can all be part of the discussion and ways forward to address public and consumer concerns related to FAW. Ultimately, understanding the economic situation facing livestock producers and reflecting on the formal and informal signals they receive regarding production practices can elevate the overall understanding regarding current and future FAW issues.
